# Assessment of Sport-Related Brain Injuries with Rapid Objective Perimetry

**DOI:** 10.3390/bioengineering13070738

**Published:** 2026-06-25

**Authors:** Bhim B. Rai, Faran Sabeti, Emilie M. F. Rohan, Joshua P. van Kleef, Corinne F. Carle, Ted Maddess

**Affiliations:** 1 John Curtin School of Medical Research, Australian National University, Canberra, ACT 2601, Australia; faran.sabeti@canberra.edu.au (F.S.); emilie.rohan@anu.edu.au (E.M.F.R.); joshua.vankleef@anu.edu.au (J.P.v.K.); corinne.carle@anu.edu.au (C.F.C.); ted.maddess@anu.edu.au (T.M.); 2 Discipline of Optometry, Faculty of Health Sciences, University of Canberra, Canberra, ACT 2617, Australia

**Keywords:** concussion, functional loss, objective perimetry, rugby, sport-related brain injury, sport-related concussion, traumatic brain injury, visual field defects

## Abstract

Sport-related mild traumatic brain injury (mTBI) or concussion is common and has long-term implications. The lack of diagnostically accurate, rapid, and easy-to-administer tests exacerbates the problem. We evaluated the objectiveFIELD Analyser^®^ (OFA^®^) for mTBI. This cross-sectional study included athletes who had sustained a concussion, and two groups of controls: the putative control group (pCG) comprising rugby players who claimed they had never had a concussion, and a non-rugby normal control group (nCG). Two OFA tests, the 8 min (OFA30) and the rapid 90 s (OFA30-12), were performed. Discrimination was performed against both the control groups using the Area Under Receiver Operating Curves (AUROC) and Hedge’s g standardised effect size. The athletes were divided into the Acute Group, with 42 athletes tested within 15.4 ± 13.6 days of mTBI, and the Chronic Group, with 23 athletes tested within 941.5 ± 769.0 days. Subjects were age-matched (22.4 ± 3.06 years). For the nCG, OFA30-12 performed better than OFA30: with Hedge’s g values of 1.22 in acute and 1.45 in chronic cases, compared with 0.93 for acute and 1.00 for chronic cases. AUROCs performed similarly. Notably, when compared with the pCG, both tests showed poorer diagnostic power. OFA perimetry showed potential as reliable, rapid test for assessing concussion. The results cannot be generalized to the first 72 h following a concussion due to insufficient data from participants assessed within this early period.

## 1. Introduction

Traumatic brain injury (TBI) is characterised by alterations in brain function or other signs of brain damage due to external forces [1]. TBI can range from mild (e.g., concussion) to severe forms, and potentially lead to long-term cognitive, physical, and emotional impairments, thereby constituting a major health problem [2]. It may severely diminish the quality of life for both, the survivors and their families, as well as the community at large [3], posing an enormous economic burden with annual costs estimated at around $400 billion globally [4]. In Australia, 190,000 to 200,000 cases of TBI occur annually, of which about 180,000 are mild, including concussion [5]. Essentially, all concussions are mild TBIs, but not all TBIs are concussions [6]. TBIs are classified based on the Glasgow Coma Scale, whereas concussions are defined by temporary changes in brain function and can occur with or without loss of consciousness [7]. These functional changes occur due to the rapid release of neurotransmitters causing ionic disequilibrium across neuronal membranes [8]. The injury causes diffuse axonal injury resulting from physical twisting and stretching of neurons, which damages the cell membrane and disrupts the axonal cytoskeleton [9]. Damage to the membranes forces the indiscriminate release of excitatory neurotransmitters, such as glutamate. This triggers an unregulated opening of ion channels, causing a massive efflux of potassium ions, and an influx of sodium and calcium ions, resulting in ionic imbalance [10].

Due to the absence of any established test or biomarker for concussion, the current diagnostic approach includes confirming the presence of a constellation of symptoms and signs after a person has experienced a head trauma. Neuroimaging is applicable in more severe TBIs, but not in concussion [11]. Functional tests are more useful in concussion, but most of the currently available ones are subjective and have their own limitations [12]. Therefore, these are not performed as often as required to account for the high variability of reports of subjective tests. Visual field defects in persons with loss of consciousness due to concussion within 30 min have been reported, although no definitive pattern has been identified [13]. Previously, we reported correlations between per-region response delays and retinal thickness in concussed persons with a multifocal pupillographic objective perimeter (mfPOP) [14], now commercially available as the objectiveFIELD Analyser^®^ (OFA^®^). One of the tests used here has been shown to be comparable to or have better diagnostic power than standard automated perimetry (SAP) in glaucoma [15] and early diabetic retinopathy [16]. The cortical basis of the responses to OFA’s transient yellow stimuli is well established [17,18] and has been used to study visual attention [19]. OFA tests have also been reported to have high diagnostic [20] and prognostic [21] power in multiple sclerosis. OFA safety and diagnostic power data have also been reported for migraine [22] and epilepsy [23]. Any established pattern of functional changes would be useful to guide the diagnosis and the severity of concussion. The current study investigated the diagnostic power of an older 8 min and a new rapid 90 s OFA test in athletes who suffered a concussion during sports in both acute and chronic cases.

## 2. Materials and Methods

### 2.1. Study Design and Ethics

This cross-sectional study was approved by the Australian Capital Territory Human Research Ethics Committee (ETH01499), and informed written consent was obtained from all the participants. The research adhered to the tenets of the Declaration of Helsinki.

### 2.2. Subjects

We collaborated with local Canberra rugby clubs and public and private physiotherapy clinics in the Canberra region to recruit athletes who had suffered concussions. We included mostly male and two female athletes who had suffered a concussion during sports (test subjects). We categorised them into two groups: the *Acute Group* with concussion occurring within 45 days before the test day, and the *Chronic Group* with concussion occurring more than 2 months before the test day. The athletes were judged to have been concussed by an accredited sports physiologist or physiotherapist on the field of play, and the TBI was considered serious enough for them to be asked to leave the field for the remainder of the day, whether or not they lost consciousness. We also recruited 2 groups of control subjects—a group of rugby players who claimed to have never had a concussion (*Putative Control Group*), and another group of non-athlete normal male individuals (*Normal Control Group*). The desired number of participants in each group was based on the power calculation to achieve an effect size of 1.4, and a power of 0.95; 12 participants were required per group.

None of the test subjects were symptomatic at the time of the study tests and were not taking any medications that would potentially influence the autonomic nervous system or pupillary functions. The participants, both test and control subjects, did not drink caffeinated beverages within 1 h before testing.

### 2.3. OFA Tests and Stimuli

**Figure 1:**
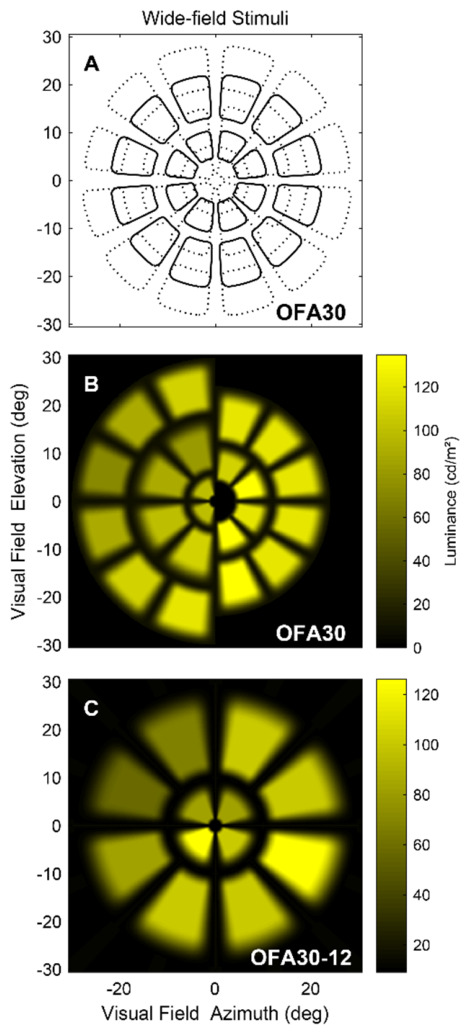
Representations of the 2 OFA stimulus types of the study. (**A**) Shows contours of the 44 regions of OFA30. In practice, the regions are never presented with overlapping stimuli. (**B**) The left and right halves of the 5 rings of OFA30 stimuli showing their relative intensities. (**C**) The stimuli of the 90 s OFA30-12 wide-field array. Stimuli are shown for the left eye, and right eye stimuli were left-right mirror symmetric.

OFA tests both eyes simultaneously and independently. It provides two key data types from the relative amplitude of pupillary constriction: changes in retinal sensitivity (both hypersensitivity and hyposensitivity) measured in decibels (db); and the response delay measured as the time-to-peak constriction in milliseconds (ms). These variables are measured at all tested visual field locations. The response delay is a unique advantage of the OFA, which is not provided by any other visual field testing device, and is crucial for the assessment of brain and neurological disorders such as multiple sclerosis [20]. Given the success of our initial study of concussion [14], we decided to explore this finding in a larger group. Two OFA tests were investigated: the 4th-generation high spatial resolution, 8 min test, OFA30 (Figure 1A,B); and the new rapid 5th-generation 90 s, OFA30-12 test (Figure 1C). Both tests assess the central 60 degrees and were preformed in random order.

To avoid confounding factors, the test room was isolated to minimize external disturbances and had dim office lighting of 100 Lux measured at the OFA device. Any vergence deficit was corrected before starting the tests. The maximal luminance of the stimuli was 288 cd/m^2^, presented on a 10 cd/m^2^ background.

### 2.4. Ophthalmic Examinations

All test procedures were performed on both putative and normal control subjects in settings similar to those described above. OFA tests were followed by other tests on the same day to avoid confounding factors affecting the OFA reports due to exposure to light from other devices. We performed 24–2 visual field testing with the Matrix perimeter (Carl Zeiss Meditec Inc., Dublin, CA, USA), measured best-corrected visual acuity (BCVA) with the Early Treatment Diabetic Retinopathy Study (ETDRS) chart, conducted slit-lamp examination including 90-dioptre bio-microscopy to rule out pupillary abnormalities, media opacities, and retinal disorders, and measured intraocular pressure (IOP) with Goldmann applanation tonometry. Macular retinal thickness posterior pole scans providing both 8 × 8 grid data and 9 ETDRS subfield data with 25 ART frames, 61 sections, and line spacing of 120 µm, and retinal nerve fibre layer (RNFL) scans were performed with a Spectralis Optical Coherence Tomography (OCT) (Heidelberg Engineering GmbH, Germany). Retinal layer thickness was calibrated by measuring the corneal curvature with an auto-refractometer (ARK-1s NIDEK Co. Ltd., AICHI Japan).

### 2.5. Analysis

OFA and other analyses employed MATLAB (2020b, The MathWorks, Natick, MA, USA). Power calculations used G*Power (version 3.1, University of Kiel). The normative models were visual field maps created by taking the median value of measures of retinal sensitivity and response delay at each visual field location of the control subject groups—the Putative Control Group and the Normal Control Group separately. All controls were males of a similar age. We felt that the subject numbers did not warrant more complex normative models. We examined Pattern Deviations (PDs) (deviations from the normative data adjusted to the 86th percentile of the field) for the per-region sensitivities and delays. In particular, we examined combined per-region scores of the sensitivity and delay PDs, which performed the best. Area Under Receiver Operating Curve plots (AUROC) were assessed for the single worst point, the mean of the worst 2, 3, and so on [24]. Each of these means for these N-worst points provided a single score for each eye. The worst two points were discarded from every field before the formation of the means. We compared the AUROCs with standardized effect sizes assessed as Hedge’s *g*, which is Cohen’s *d* corrected for different group sizes to make them comparable. We have reported similar comparisons of AUROCs and *g* for similar N-worst analyses in AMD [25]. The conventions for effect size are illustrated in Table 1.

## 3. Results

### 3.1. Demographic Characteristics

The study included a total of 65 test subjects who suffered a concussion, 31 subjects in the Normal Control Group, and 14 athletes in the Putative Control Group. The Normal Control Group did not play sports and claimed to have had no concussions. The Putative Control Group comprised rugby players who also claimed to have had no concussions. The Putative Control Group comprised 14 male rugby players aged 22.3 ± 2.37 years. The Normal Control Group comprised 31 subjects—17 subjects aged 22.4 ± 4.11 years for OFA30-12, and 14 subjects aged 22.2 ± 3.60 years for OFA30. There were two sports concussion groups. The Acute Group included 42 subjects who had their concussion within 41 days of testing—20 of them within 36 days and 22 of them within 21 days (range: 3–41 days, mean ± SD, 15.4 ± 13.6 days). The Chronic Group consisted of 23 subjects who had their concussion between 69 and 2961 days (941.5 ± 769.0) before testing. Because 45 days neatly split these groups, it was used as a cutoff. All but two of the subjects were males, rugby players with a mean age of 22.4 ± 3.06 years (range: 18-33 years).

### 3.2. Diagnostic Power of OFA Tests

Interestingly, the outcomes depended critically upon which control group was used: the Putative Control Group (Table 2) or the Normal Control Group (Table 3). The data are based on the combined per-region scores of the sensitivity and delay PDs. Here, we report outcomes for the mean of the worst six or nine regions per field (most deviating from normal). When the Normal Control Group was used for comparison, the rapid OFA30-12 showed greater diagnostic power, with AUROC Values falling within the mean + standard errors (SEs), and often yielding effect sizes of >1.2 (‘Very Large’) for Hedge’s *g*. Thus, it performed better than the longer OFA30 test for Hedge’s *g* values of 1.22 in acute and 1.45 in chronic cases c.f., compared with 0.93 in acute and 1.00 in chronic cases. The AUROC values (±95% confidence limit (CL)) were also superior for OFA30-12 for both acute and chronic concussion. When compared with the Putative Control Group, the OFA30-12 and OFA30 tests showed poorer diagnostic power. However, in both scenarios, the diagnostic power of the OFA30-12 test was superior to that of the OFA30 test with respect to both AUROC and effect size. The best performance was obtained with the mean of the nine regions showing the greatest deviations from normal. Better OFA performance with AUROC and effect size was obtained for the Chronic Group than for the Acute Group for both OFA30-12 and OFA30 tests. The values of Hedge’s *g* were in accord with what would be expected from the AUROCs [26]. It is worth noting that when the 95% CLs are considered, most of the AUROC values in the bottom three rows of Table 2 were not significantly different (at *p* = 0.05) from chance performance.

## 4. Discussion

There has been a growing concern about both the short-term impacts and long-term consequences of concussions. In the short term, premature return to play may increase the risks of adverse outcomes and impact the individual more severely, causing permanent damage [27]. In the long term, chronic concussion can result in physical, cognitive, and emotional impairments. Repeated trauma or post-concussion syndrome may lead to lasting symptoms such as chronic headaches, memory loss, depression, and an increased risk of neurodegenerative diseases and chronic traumatic encephalopathy [28]. Emphasis has been placed on the accurate diagnosis and management of concussions in sports [29]. However, diagnosing and grading the severity of concussion remain challenging because symptoms can be subtle, delayed, or inconsistent, and there are no definitive guidelines for the confirmatory diagnosis of concussion [30]. There are also no reliable radiological or laboratory investigations that assist with the diagnosis of concussion [31]. Other challenges include the fact that a concussion is an invisible injury that cannot be seen on any medical imaging, patients may not report symptoms, or they may downplay their injury, especially during sports. Cognitive and emotional symptoms are often the most telling, but can be misinterpreted or overlap with other conditions, such as anxiety, depression, or the influence of the game [32]. Therefore, it is imperative to establish standard methods and set guidelines for the diagnosis of concussion on playing fields. A concussion is primarily a functional impairment resulting from biochemical and neurometabolic changes in the brain, rather than gross structural damage that can be visible on standard imaging such as computed tomography (CT) or magnetic resonance imaging (MRI) [33]. Therefore, any functional test providing reliable reports is desirable. Visual field dysfunction has been reported in war fighters following blast and non-blast mild TBI using subjective perimeters [13]. In this study, we validated an objective perimeter, OFA, in the assessment of concussion.

With the growing evidence that the pupillary function for transient onset luminance stimuli is controlled by higher cortical centres [34,35]. OFA stimuli have been deliberately designed to stimulate responses from cortical pathways [17,18]. Not surprisingly, then, OFA has demonstrated utility in migraine [22], epilepsy [23] and visual attention [19]. The clinical utility of OFA30-12 has been demonstrated in multiple sclerosis [20,21]. We have shown that pupil-based functional testing can be useful in diagnosing acute concussion [18]. In the current study, OFA showed strong diagnostic power with respect to the AUROC and Hedge’s *g* effect size for both acute and chronic concussion. For comparison with studies reporting *p*-values, we note that an effect size of 1.4 means that detecting the difference between two independent groups at *p* = 0.01 and a power of 0.99 requires only 24 persons per group, and 17 persons per group at *p* = 0.05 and a power of 0.99. The Putative Control subjects who claimed never to have had any concussion might have had some level of concussion, but it was not noticed, not diagnosed, or not reported. This assumption is supported by studies showing a high prevalence of unreported and unrecognized concussions, with some players intentionally not reporting injuries for fear of being sent off the field or losing their contracts as players [36]. Besides the unreported cases, the *tip of the iceberg phenomenon* is compounded by sub-concussive hits, lack of education on sport-related head injuries and their sequelae, and pressure to play sport [36]. A study on diagnosed, unreported, and unrecognised concussions among community rugby players reported the prevalence rates of 66.5%, 32.4%, and 42.2%, respectively. That study also reported that the players with diagnosed concussions had a 7.2-fold higher prevalence of nondisclosure, and that a longer playing history was related to a greater nondisclosure [37]. The poor diagnostic results of OFA in the Putative Control Group might suggest that its members had cumulative damage, even though they claimed not to have had a concussion serious enough to be asked to leave the field. All these findings suggest that the test data must be compared with non-athlete control data for accuracy. Secondly, these findings also suggest that OFA has demonstrated strong efficacy in differentiating athletes with concussion and even sub-clinical concussion from non-concussion athletes or normal subjects. The OFA30-12 produced usable diagnostic power [38] when the Normal Control Group data were used. In a comparative review of 44 functional and structural tests from 23 studies for the detection of early diabetic eye damage, OFA had the best diagnostic power [39], confirming the utility of the pupil response in detecting early neural changes in the retina. The OFA is known to diagnose early-stage or even pre-clinical retinal diseases, such as diabetic retinopathy [40] and early age-related macular degeneration [41]. With high efficacy in diagnosing functional loss, the OFA may be valuable in assessing concussion.

Another significant finding from this study is that the OFA30-12 performed the best among the test subjects with chronic concussion, showing a higher Hedge’s g effect size compared to that of the subjects with acute concussion. It is known that approximately 30% of subjects with concussion progress to a chronic state known as Persistent Post-Concussion Symptoms (PPCS), also called Post-Concussion Syndrome, which is associated with functional compromise and can be highly disruptive to the daily lives of the affected individuals [42]. Due to injury to the brain and the resulting autonomic and vascular dysfunction, the brain’s ability to automatically regulate blood flow becomes impaired, leading to reduced cerebral perfusion and cumulative neural damage [43]. Functional MRI scans show that persons with chronic concussion display atypical activation patterns—the brain must work much harder and use alternative pathways to complete basic memory or attention tasks [44]. In chronic concussion or PPCS, functional overlay refers to the way non-structural, non-neurological factors—such as headache, sleep disturbance, vestibular dysfunction, anxiety, and psychological distress—interact with the original injury and amplify symptoms, leading to functional compromise far beyond what the initial concussion alone would cause. [45]. Therefore, considering the underlying pathophysiology and the interplay among these mechanisms, it is reasonable to conclude that our novel test, OFA, demonstrates greater diagnostic power in individuals with chronic concussion.

The application of OFA to concussion is supported by our work on epilepsy [23], which is clearly a cortically mediated disease. In that study, regional field sensitivities were increased in people with generalised epilepsy (3.80 ± 1.43 dB compared to controls). By contrast, an extra delay of 24.9 ± 10.2 ms was seen in the time-to-peak of responses in people with focal epilepsy. The same group of subjects was investigated on the same day, examining *alphaband gain* [46]. That alpha-wave-based electroencephalogram (EEG) measure also distinguished generalized and focal epilepsy, seemingly reinforcing the OFA finding. That is interesting because alpha has recently been shown to be related to predictive encoding [47]. Incorrect percepts have been proposed to be generated by a mismatch between visual perceptual priors and incoming sensory information [48]. Related perceptual faults are suspected to underlie some aspects of psychoses [49], as well as hallucinations and synaesthesia [50]. Abnormal alpha has been reported for schizophrenia [51], schizoaffective disorder [52], and attention deficit hyperactivity disorder (ADHD) [53]. Thus, the link between alphaband gain and OFA suggests that the OFA has a role in these types of disorders.

The other advantage of utilising OFA is its objective evaluation of function, providing more repeatable and reliable results compared with all other visual field test methods [54]. The OFA provides a rapid testing of both eyes in under 90 s as previously demonstrated in an AMD study [25], and can similarly evaluate athletes with suspected concussion. Besides the AUROC and effect size, another parameter that can be assessed in athletes with concussion is retinal structure changes, including thickness, as reported by some studies [14,18,55,56]. Specifically, there is thinning of the retinal nerve fibre layer (RNFL), which has been correlated with the cerebral white matter loss and neurodegeneration [57]. The RNFL thinning in mild TBI is associated with visual field defects [56]. Previously, one study reported that retinal structural changes measured by OCT correlated with the functional changes measured by the OFA in mild TBI [14].

Better OFA performance was obtained for the Chronic Group than for the Acute Group, which may reflect the cumulative damage acquired over many years or the delayed threshold for neurodegeneration, as explained above, that is sufficient to impact pupillary responses. This ability of OFA can be utilized for long-term follow-up of the subjects who have suffered a concussion.

There are different standardized clinical tests used as sideline tools in the assessment and management of concussions. These include the Sport Concussion Assessment Tool 6 (SCAT6) [58], Standardised Assessment of Concussion (SAC) [59], and Concussion Recognition Tool 6 (CRT6) [60] tests. Additionally, there are vestibular, ocular, and balancing screening tests, which include Vestibular/Ocular Motor Screening (VOMS) [61], Balance Error Scoring System (BESS) [62], and King–Devick (K-D) [63] tests. The SCAT6 test is designed for healthcare professionals, including general practitioners and medical doctors, and requires additional training to perform the test correctly [58]. The SAC test consists of different assessment criteria, such as orientation, immediate memory, neurological screening, concentration, and delayed recall, each with different score levels that add up to a total score. Even experienced clinicians find it challenging to administer this test accurately [59]. The CRT6 test is primarily for the general population. It includes red flags, signs, and symptoms to identify a suspected concussion, but it is frequently associated with subjective error [60]. The VOMS test is reported to have excellent internal consistency and moderate-to-good test–retest reliability. However, it has a very high false-positive rate of 21.9%, limiting its effective utility [61]. The BESS test is also a subjective test, so it is not possible to determine if the athletes perform poorly on purpose [62]. The K-D test has acceptable diagnostic value only at certain post-concussion periods, but is not adequate at other times. It should be used with caution, with repeated administration required, as it is known to exhibit practice effects [63]. Overall, most of the currently available tests utilize subjective criteria for the diagnosis of concussion and are time-consuming as well. Some tests are effective only at specific time positions after head injury, while the results of others are impacted by repeated administration due to the learning or practice effects. On the other hand, OFA is an objective and easy test capable of assessing an athlete in 90 s and has low test–retest variability [15].

The current study is the first to evaluate the diagnostic power of the rapid fifth-generation (OFA30-12) compared with the longer fourth-generation (OFA30) test. Our study is limited by a small number of test subjects, especially in the Putative Control Group, which contained only 14 participants. However, this small number of participants in this group is acceptable because the observed effect size was 1.45, and lowering the power to 0.95 indicated that only 12 persons per group were required. Hedge’s *g* and AUROC provide clinically meaningful information beyond *p*-values. The patients were categorized into acute and chronic groups arbitrarily, which does not comply with the clinical classification. We did not have cases within the first three days after concussion, and therefore, we could not report on how the OFA performed in athletes at such acute stages. Therefore, the results of the current study cannot be generalized to the first 72 h following a concussion due to insufficient data from participants assessed within this early time period. A larger study with more participants, including more acute cases within the first 72 h after concussion, is needed for further evaluation.

## 5. Conclusions

Rapid objective perimetry, OFA30-12, performed well and produced usable diagnostic power in assessing mild TBI or concussion. The OFA30-12 showed potential as an objective tool for concussion assessment after the first 72 h. Further studies with larger numbers of subjects, including athletes with immediate head trauma, are needed to evaluate its utility in the immediate post-injury period and for monitoring recovery.

## Figures and Tables

**Table 1:** Standardised Effect size levels for Hedge’s g.

Effect Size	Hedge’s G
SMALL	0.2
MEDIUM	0.5
LARGE	0.8
VERY LARGE	1.2
HUGE	2.0

**Table 2:** Diagnostic power of OFA in Athlete Controls.

Test Types	AUROC^+^ (*N* = 6)	AUROC^+^ (*N* = 9)	Hedge’s *G* (*N* = 6)	Hedge’s *G* (*N* = 9)
**OFA30-12—**ACUTE	73.5 ± 12.0	74.0 ± 12.1	0.82 ± 0.13	0.79 ± 0.13
**OFA30-12—**CHRONIC	65.1 ± 13.7	63.2 ± 13.8	0.32 ± 0.07	0.27 ± 0.06
**OFA30—**ACUTE	48.9 ± 8.1	49.4 ± 8.2	0.15 ± 0.02	0.14 ± 0.02
**OFA30—**CHRONIC	57.4 ± 13.9	57.5 ± 14.1	0.22 ± 0.05	0.21 ± 0.05

^+^ Area Under Receiver Operating Curve plot; N refer to number of worst cases; Acute and Chronic refer to recent (within 45 days) and much earlier (longer than 2 months) concussions. Errors are 95% Confidence Limit (CL).

**Table 3:** Diagnostic power of OFA in Non-Athlete Controls.

Test Types	AUROC^+^ (*N* = 6)	AUROC^+^ (*N* = 9)	Hedge’s *G* (*N* = 6)	Hedge’s *G* (*N* = 9)
**OFA30-12—**ACUTE	79.4 ± 8.1	77.9 ± 8.8	**1.22** ± 0.12	1.11 ± 0.12
**OFA30-12—**CHRONIC	82.6 ± 10.2	82.9 ± 9.8	**1.45** ± 0.18	**1.44** ± 0.17
**OFA30—**ACUTE	76.0 ± 10.8	75.6 ± 11.0	0.93 ± 0.13	0.94 ± 0.14
**OFA30—**CHRONIC	78.1 ± 10.3	78.4 ± 10.3	1.00 ± 0.13	1.00 ± 0.13

^+^ Area Under Receiver Operating Curve plot; N refer to number of worst cases; Acute and Chronic refer to recent (within 45 days) and much earlier (longer than 2 months) concussions. Values in bold are ≥1.2, i.e., very large effect size (Table 1). Errors are 95% Confidence Limit (CL).

## Data Availability

Data available on request due to restrictions.
